# Spontaneous Neural Dynamics and Multi-scale Network Organization

**DOI:** 10.3389/fnsys.2016.00007

**Published:** 2016-02-09

**Authors:** Brett L. Foster, Biyu J. He, Christopher J. Honey, Karim Jerbi, Alexander Maier, Yuri B. Saalmann

**Affiliations:** ^1^Department of Psychology, Stanford UniversityCA, USA; ^2^Laboratory of Functional and Molecular Imaging, National Institute of Neurological Disorders and Stroke, National Institutes of HealthMD, USA; ^3^Department of Psychology, University of TorontoON, Canada; ^4^Department of Psychology, University of MontrealQC, Canada; ^5^Department of Psychology, Vanderbilt UniversityTN, USA; ^6^Department of Psychology, University of Wisconsin - MadisonWI, USA

**Keywords:** resting-state fMRI, electrocorticography (ECoG), brain networks, connectivity, neural dynamics

## Abstract

Spontaneous neural activity has historically been viewed as task-irrelevant noise that should be controlled for via experimental design, and removed through data analysis. However, electrophysiology and functional MRI studies of spontaneous activity patterns, which have greatly increased in number over the past decade, have revealed a close correspondence between these intrinsic patterns and the structural network architecture of functional brain circuits. In particular, by analyzing the large-scale covariation of spontaneous hemodynamics, researchers are able to reliably identify functional networks in the human brain. Subsequent work has sought to identify the corresponding neural signatures via electrophysiological measurements, as this would elucidate the neural origin of spontaneous hemodynamics and would reveal the temporal dynamics of these processes across slower and faster timescales. Here we survey common approaches to quantifying spontaneous neural activity, reviewing their empirical success, and their correspondence with the findings of neuroimaging. We emphasize invasive electrophysiological measurements, which are amenable to amplitude- and phase-based analyses, and which can report variations in connectivity with high spatiotemporal precision. After summarizing key findings from the human brain, we survey work in animal models that display similar multi-scale properties. We highlight that, across many spatiotemporal scales, the covariance structure of spontaneous neural activity reflects structural properties of neural networks and dynamically tracks their functional repertoire.

## Spontaneous brain activity

As an organ in perpetual action, the human brain consumes 20% of the body's metabolic budget, despite constituting only 2% of body weight (Raichle, 2010). This metabolic consumption supports both spontaneous processes, which occur independent of the immediate environment, as well as processes that are coupled to the external world. In systems neuroscience, the spontaneous component of brain activity has often been viewed as a kind of noise: an uninformative nuisance variable that should be controlled for in experimental design and removed during data analysis. However, it is clear that the anatomical structure and physiological processes of neurons and surrounding tissue impose constraints on the spatial and temporal properties of both spontaneous and task-related neural activity. Thus, spontaneous activity patterns should reflect these spatiotemporal constraints.

A wealth of empirical data across different levels of investigation supports the notion that spontaneous neural activity is functionally relevant. Most recently, studies of the organization of spontaneous hemodynamic activity in the human brain using functional magnetic resonance imaging (fMRI) have provided reliable maps of covariation in cortical and subcortical activity (Power et al., 2011, 2014; Yeo et al., 2011; Buckner et al., 2013). This approach can extract and parcellate sets of covarying regions (“functional networks”) that match the sets of regions co-active during task conditions. For this reason, and because the covariance analyses make novel predictions of previously underappreciated functional divisions, there is now widespread interest in the spatial organization and temporal dynamics of spontaneous brain activity (Buckner et al., 2013; Hutchison et al., 2013; Power et al., 2014).

In this Review, we outline existing evidence that brain networks identified via resting-state fMRI (rsfMRI) have clear electrophysiological correlates in the human brain. We highlight the most popular techniques used to study correlated patterns of spontaneous neuronal activity in the resting-state, and examine the contribution of these techniques to understanding the origin and implications of spontaneous cortical activity. Next we discuss how similar conceptual and analytical methods have previously been applied to neuronal and population scale electrophysiology. We highlight similar observations across studies of spontaneous connectivity (at the regional/macro scale) and studies of spontaneous noise correlations (in activity at the cellular/micro and circuit/meso-scale). Despite several orders of magnitude of difference in spatial scale, recent empirical work has highlighted that spontaneous brain activity can capture important structural and functional properties of local and global neural systems, consistent with the view that the statistical properties of spontaneous activity reflect anatomical and physiological constraints of organizational principles within the central nervous system.

## Correlated spontaneous hemodynamics

Hemodynamic activity in the mammalian brain displays a sensitive and complex relationship to local electrophysiological activity (Logothetis, 2012). Utilizing the compensatory delivery of oxygenated hemoglobin to sites of elevated neural activity, blood oxygenation level dependent (BOLD) fMRI provides an indirect and non-invasive whole brain measurement of large-scale neural activity (Logothetis and Wandell, 2004). As hemodynamic responses are often consequent to local neural events, and modulated by cardio-vascular physiology, BOLD fMRI activity is a delayed, and slow time varying (<1 Hz) signal. Despite these limiting properties, spontaneous BOLD fMRI displays ongoing large amplitude signal variations, even when subjects are not performing any explicit task (e.g., awake, with eyes closed or fixating). Indeed, the spontaneous fluctuations in BOLD activity can be larger in magnitude than sensory-evoked responses (Raichle, 2010).

Beginning with observations by Biswal et al. (1995), empirical studies of spontaneous BOLD fMRI have revealed spatial covariance across cortical and subcortical structures that often conforms to previously established functional brain networks. For example, rsfMRI signal covariance can be used to parcellate sensorimotor systems (van Den Heuvel and Hulshoff Pol, 2010; Asemi et al., 2015), higher order associative fronto-parietal networks (Vincent et al., 2006, 2008), thalamic nuclei (Zhang et al., 2008; Fan et al., 2015), and cerebellar cortex (Buckner et al., 2011). In particular, studies quantifying correlational structures in spontaneous BOLD signals during non-task “resting-states” have grown exponentially over the past 20 years, resulting in detailed parcellations of functionally separated cortical networks in the human brain (e.g., Yeo et al., 2011; Power et al., 2014).

A key factor in the continued and expanding interest in studying spontaneous fMRI signals has been the striking correspondence between resting-state functional networks and previously established maps of functional co-activation accumulated by cognitive neuroimaging (e.g., Smith et al., 2009; Cole et al., 2014). This correspondence opens the exciting possibility that correlational structures in spontaneous fMRI data provide an economical and non-invasive assay of large-scale functional network organization (Power et al., 2014). As noted above, a key biological determinate/constraint of the structure of spontaneous activity are the ontogenetic factors defining anatomical structure in the human brain (Bressler and Tognoli, 2006). However, rsfMRI connectivity displays interesting departures from anatomically prescribed organization (Honey et al., 2009), as some regions, linked through poly-synaptic pathways rather than direct connections, display strong spontaneous correlation (e.g., Buckner et al., 2013). It is in this regard that rsfMRI, and spontaneous neuronal activity in general, reflects both constraints imposed by anatomy, and constraints imposed by the context and history of neural events that sculpt functional networks.

As a putative assay of functional brain network organization, and as a non-invasive technique relying on basic physiological properties of the nervous system, rsfMRI can also be employed for comparative studies across mammalian species (Hutchison and Everling, 2012; Buckner and Krienen, 2013), across clinical populations (Buckner et al., 2013), and within subjects to assess the influence of learning (Lewis et al., 2009; Guidotti et al., 2015). However, rsfMRI investigations were met with initial skepticism (Buckner and Vincent, 2007; Morcom and Fletcher, 2007) due to technological challenges (Birn et al., 2006; Power et al., 2012; Van Dijk et al., 2012), whose resolution defines important milestones of the field (Power et al., 2014). Of particular note is that several researchers have highlighted the uncertain interpretation of rsfMRI because of the unstructured nature of resting-state cognition (Morcom and Fletcher, 2007), as well as the complex relationship between BOLD signal fluctuations and ongoing electrophysiological activity (Leopold and Maier, 2012). Below, we focus on the literature assessing the electrophysiological correlates of rsfMRI in human and non-human primates. We then highlight how spontaneous electrophysiological data from the micro- and meso-scopic (neurons and circuits) levels display similar organizational principles found on the macroscopic (whole brain) level.

## Electrophysiology of correlated spontaneous hemodynamics

Human and non-human primate rsfMRI has suggested that networks of cortical and subcortical regions can be reliably identified by examining the covariance structure of spontaneous hemodynamics (e.g., Power et al., 2011; Yeo et al., 2011; Hutchison and Everling, 2012; Power et al., 2014). Prior to large-scale efforts to map human brain connectivity with rsfMRI (Van Essen et al., 2013), a number of criticisms were raised regarding the neural authenticity of slow (<1 Hz) varying hemodynamics (Snyder and Raichle, 2012). Identifying the electrophysiological correlates of this hemodynamic activity is therefore important; both for providing a neuronal basis to rsfMRI-defined networks as well as for elucidating mechanisms that may drive rsfMRI dynamics. As discussed below, although experimentally challenging, invasive techniques provide rich spatial, temporal, and spectral information for exploring rsfMRI correlates (for reviews of non-invasive findings see Engel et al., 2013; Schölvinck et al., 2013; Hall et al., 2014). We begin by outlining the most common analytical techniques for quantifying inter-regional coupling in spontaneous electrophysiological data.

### Approaches to measuring coupling in spontaneous brain activity

A standard approach for characterizing the spatial organization of spontaneous neural activity is to compute Pearson's correlation (r) between time series of activity, i.e.,

$$\begin{array}{l} r=\frac{\frac{1}{n}\overset{n}{\underset{t=1}{\sum }}\left(X_{t}-{\bar{X}}_{t}\right)\cdot \left(Y_{t}-{\bar{Y}}_{t}\right)}{\sqrt{\frac{1}{n}\overset{n}{\underset{t=1}{\sum }}{\left(X_{t}-{\bar{X}}_{t}\right)}^{2}}\cdot \sqrt{\frac{1}{n}\overset{n}{\underset{t=1}{\sum }}{\left(Y_{t}-{\bar{Y}}_{t}\right)}^{2}}} \end{array}$$

where *X*_*t*_ and *Y*_*t*_ are two times series of interest, with $\bar{X}$_*t*_ and $\bar{Y}$_*t*_ being their respective means. Thus, *r* measures the covariance between two signals normalized by the individual variance of each signal, a ratio that is bounded to the range [−1, 1]. Algebraic reformulation also depicts *r* as the mean of the dot product of standardized scores (z-score) for *X* and *Y* (i.e., the dot product moment correlation coefficient; (Lee Rodgers and Nicewander, 1988)). As a linear parametric statistic, *r* assumes *X* and *Y* to be continuous variables that are normally distributed and that, if related, share a linear association. In addition, *X* and *Y* should not contain univariate or bivariate outliers, and display homoscedasticity (i.e., homogeneous variance of least-squares residuals; Pernet et al., 2012). For rsfMRI data, correlated variables are typically the preprocessed fMRI BOLD time series from regions of interest such as spatially separated voxels or voxel-clusters. For electrophysiological data, which contains greater temporal resolution, first and second order spectral features, including amplitude and phase, are commonly studied. For electrophysiological data, correlational analyses focus on either the raw, broadband time series, band limited time series, or the amplitude of band-limited time series.

Typically, correlation of broadband time series is not desirable as this signal conflates, non-uniformly, the spectral content of electrophysiological signals (i.e., due to their high power relative to higher frequencies, the lowest frequencies dominate the signal). A more common approach is to study correlations within and across band limited frequency ranges. Studies of band limited correlations typically focus on the amplitude or power within the selected frequency band. While a number of different time-frequency approaches can be employed toward this aim (Engel et al., 2013), the procedure adopted by numerous studies seeks to isolate a frequency range of interest through filtering or convolution, and to extract the instantaneous (i.e., sample-wise) time-varying amplitude of the band limited signal. For example, an experimenter might seek to study the correlation between two regions in the alpha band range between 7 and10 Hz. First, the spontaneous raw time series of interest is band pass filtered between 7 and 10 Hz, using an appropriately parameterized filter. Next, one can obtain the instantaneous amplitude signal of this band-limited data by applying a Hilbert transform to the filtered time series to compute the analytic signal

$$\zeta_{t}=X_{t}+iH(X_{t}).$$

**Figure 1:**
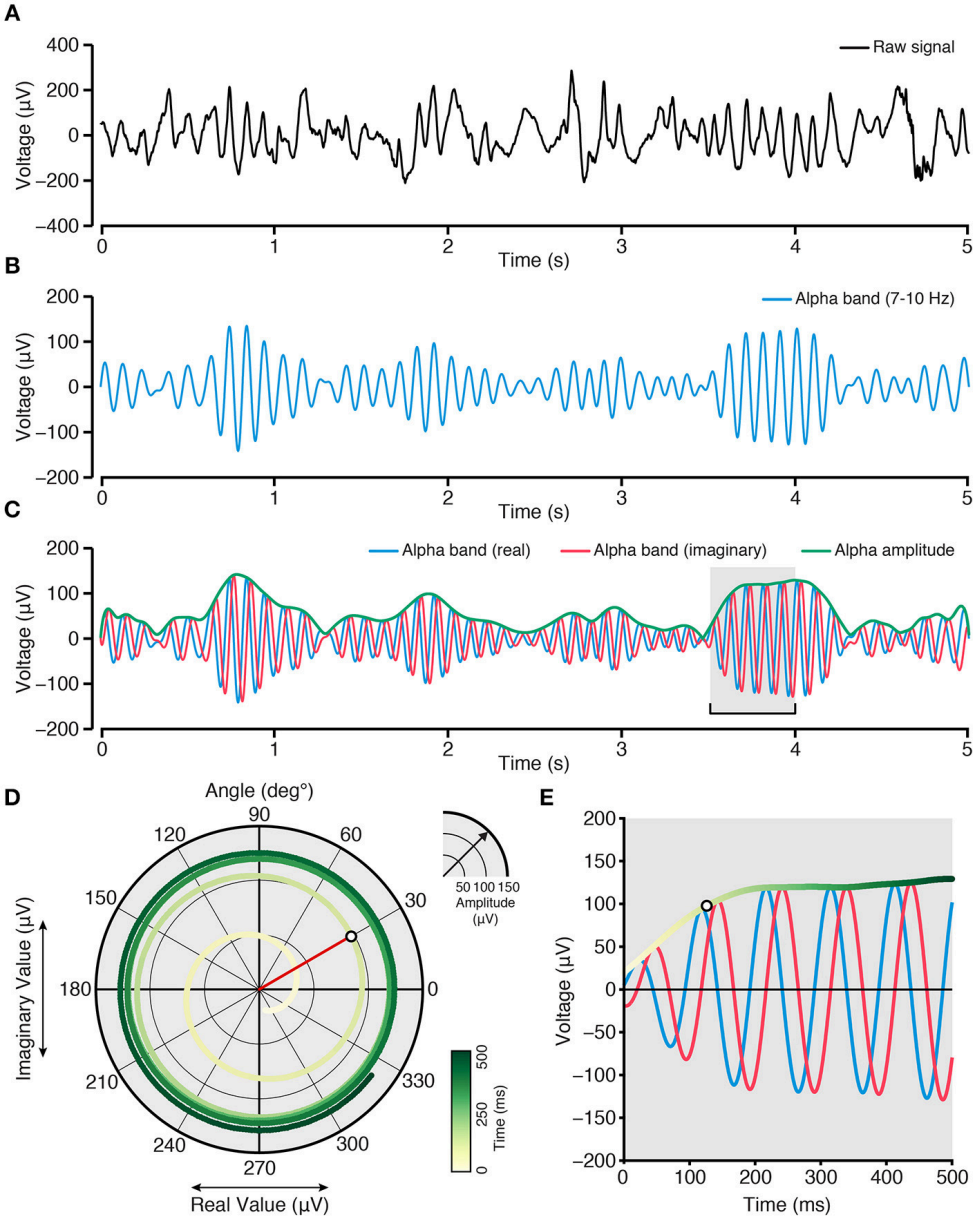
**Quantifying band limited amplitude for correlation analysis of spontaneous data**. **(A)** Raw spontaneous ECoG time series (5 s). **(B)** Spontaneous alpha band limited time series extracted from **(A)**, by a 7–10 Hz band pass filter. **(C)** Analytic signal of **(B)**, obtained via a Hilbert transform, showing the real component (blue), imaginary component (red), and amplitude/envelope (green). The real component is the original band pass signal from **(B)**, and the imaginary component is a 90° rotated instantiation of **(B)**. The amplitude/envelope (green) is the absolute value of the analytic signal (i.e., a complex valued time series with real and imaginary parts). **(D)** Complex plane showing the temporal evolution (yellow-green shading) of the analytic signal [data comes from window highlighted with gray in (**C**)]. Over time, each observation (sample) takes a coordinate location in the complex plane given the real (x-axis) and imaginary (y-axis) values at that time point [example time point with white fill color highlighted in **(D,E)**]. For any given time point, the amplitude/envelope of the signal in the complex plane is defined by the vector length extending from the central zero axes to the real/imaginary coordinate (red line). For example, as time evolves in **(D**; progress toward dark green) the trajectory of values spirals out radially, increasing the vector distance from the origin: this reflects an increase in alpha band amplitude as shown in **(E)**. Phase can also be obtained by identifying the angular position of the amplitude vector at each time point (red vector shown has a phase angle of 30°). Other time frequency methods (filter-Hilbert approach shown here) can be employed, such as Wavelet convolution to achieve comparable results (Bruns, 2004; Cohen, 2014).

The alpha band analytic signal (ζ_*t*_) is a complex valued time series (also obtainable via wavelet convolution (Bruns, 2004)), where the real component (*X*_*t*_) is the original band passed signal, and the imaginary component (*iH*(*X*_*t*_) is the Hilbert transform of (*X*_*t*_), which corresponds to a 90° rotation of (*X*_*t*_). It is important to note the time-frequency uncertainty produced by time series filtering/convolution, which make instantaneous time series only “estimates” of the band limited signal. Basic trigonometric functions can then be applied to the analytic signal to identify the instantaneous amplitude and phase of the band passed signal. To obtain the instantaneous amplitude (*a*_*t*_), also termed the signal envelope, the absolute value of the complex analytic signal is taken at each time point. For complex numbers, the absolute value is the length of the vector between the origin and the coordinate of the real and imaginary values in the complex plane (following Pythagoras' theorem, see Figure 1)

$$\begin{array}{l} a_{t}=\sqrt{X_{t}^{2}+H{\left(X_{t}\right)}^{2}}. \end{array}$$

**Figure 2:**
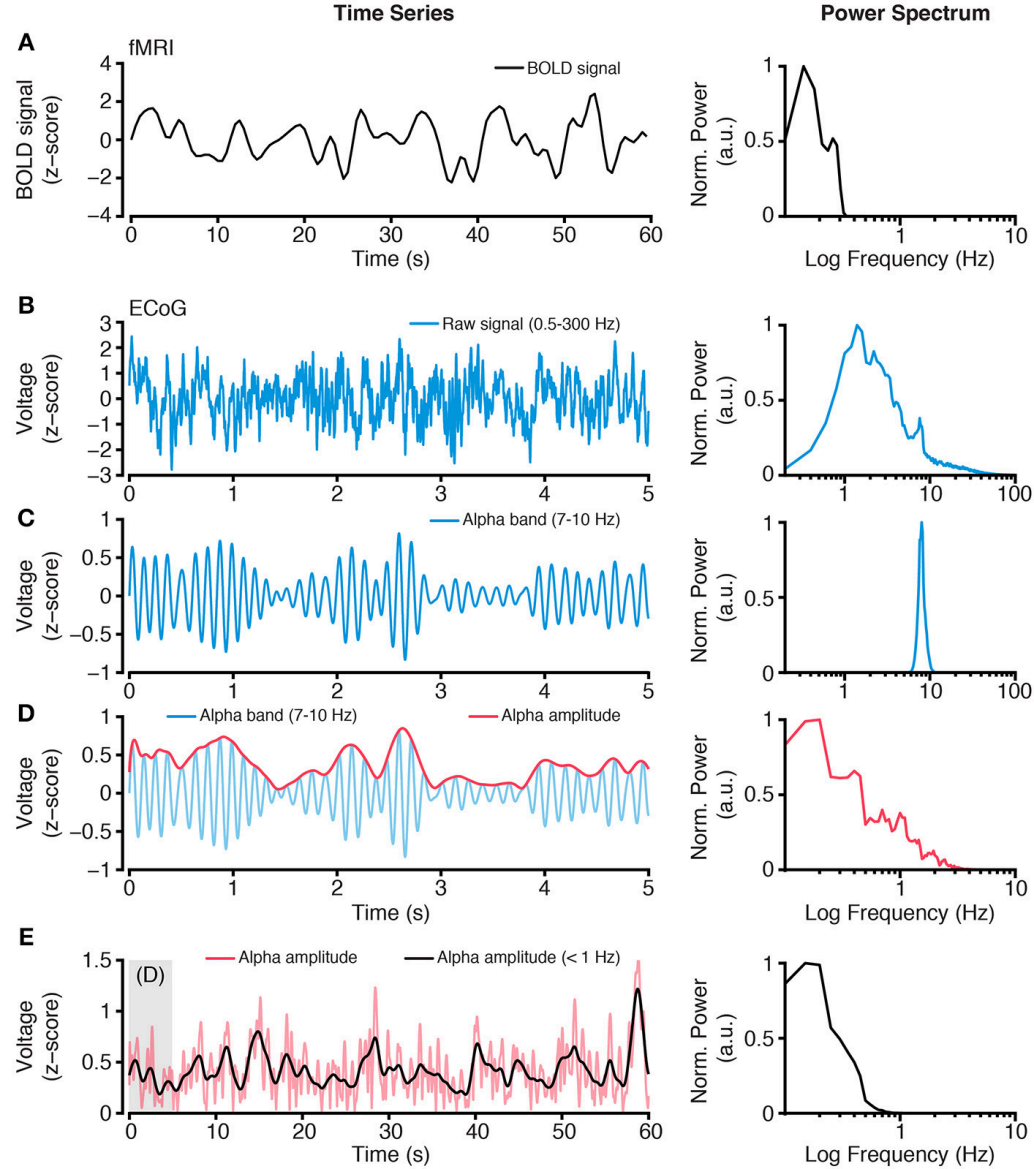
**Extracting slow time scale variability of rapid electrophysiological dynamics. (A)** BOLD fMRI activity is a slow time varying signal, due to its hemodynamic basis. Plot shows an example time course of BOLD fMRI activity (left) and the power spectrum of this signal (right). Together these plots highlight the low frequency (<1 Hz) content of BOLD fMRI. **(B)** In contrast, electrophysiological activity, such as ECoG, has a wide spectral content. However, standard ECoG recordings apply a high-pass filter limiting the study of ultra slow time scales (but see Palva and Palva, 2012). Plots show a raw ECoG time series (left), with a band pass range 0.5–300 Hz, and its power spectrum (right). The power spectrum shows a lack of power at lower frequencies (<1 Hz), owing to the recording filters, and a progressive decrease in power for higher frequencies, as commonly observed. **(C)** To study frequency specific activity patterns and inter-regional correlations, raw time series are filtered to isolate the frequency range of interest. Plots show an alpha range filtered time series (left), with a band pass of 7–10 Hz, and its power spectrum (right). The isolated alpha band activity is a rapid time varying signal, however the amplitude of alpha activity shows a slower rate of change. **(D)** Plots show the amplitude (red, left) of the alpha band time series from **(C)** and its power spectrum (right). Because the alpha band amplitude time course is not subject to the filtering of the recorded raw signal, it can contain lower frequency spectral content. **(E)** Typically, BOLD fMRI activity >1 Hz is excluded because it contains a number of non-hemodynamic artifacts (Power et al., 2014). To more closely align the time scales of hemodynamic and electrophysiological activity, the alpha band amplitude signal can be low pass filtered <1 Hz, to obtain a time series with similar spectral content as BOLD fMRI. Plots show the time course of alpha band amplitude (red) and its low pass filtered form (black; left). The power spectrum (right) of the slow time varying alpha band amplitude shows similar spectral content as the BOLD signal. As reviewed, this approach of focusing on slow time scale modulations of higher frequency activity provides a successful means of comparing hemodynamic and electrophysiological activity. Importantly, this approach can be applied to any electrophysiological frequency range of interest, and allows comparison across frequencies (Nir et al., 2008; Wang et al., 2012; Foster et al., 2015). All power spectra are calculated from extended time series from which example epochs are shown, and are normalized to the spectral maxima, with frequency shown on a log scale.

Instantaneous amplitude *a*_*t*_, in this example, reflects the sample-wise estimate of alpha band amplitude, which can be squared to obtain signal power. The resultant time series (i.e., the time-varying amplitude or power from two separate recording locations) can then be used as the signals to be correlated. Importantly, the amplitude time series constitutes a signal with its own spectral properties (Figure 2), which may contain both slow and fast dynamics (Leopold et al., 2003; Foster and Parvizi, 2012; Honey et al., 2012). This observation is important when considering the differences in time scale between hemodynamic (~0.01–1 Hz) and electrophysiological (~0.01–300+ Hz) data, and the endeavor to capture electrical correlates of hemodynamic activity. As detailed below, one approach is to extract the amplitude of a higher frequency band-limited signal across time, and to then measure slow-varying changes in this amplitude time series. The resultant time scale of slow varying changes (<1 Hz) in higher frequency neuronal activity (>1 Hz) matches that of the fMRI BOLD response (Nir et al., 2008; Ko et al., 2011; Wang et al., 2012; Foster et al., 2015), thus bridging hemodynamic and electrophysiological data (Figure 2). Another approach is to use the low-frequency raw-filtered electrophysiological signal (He et al., 2008b; Pan et al., 2013).

In addition to *a priori* seed based analysis (i.e., correlation between selected regions/electrodes), more data driven approaches such as independent components analysis (ICA) are commonly applied to spontaneous neural data (Cole et al., 2010). These unsupervised approaches seek to quantitatively identify unique spatio-temporal patterns of covariation, i.e., putative networks, without *a priori* selection of brain anatomy. ICA has been successfully applied to rsfMRI data (Cole et al., 2010), and to resting-state MEG/EEG data (Schölvinck et al., 2013; Hall et al., 2014). The variability and sparsity of spatial sampling in most invasive electrophysiological studies has limited the utility of ICA based methods, and they will not be discussed in detail here.

As noted above, instantaneous phase can also be extracted and “correlated” between two regions of interest. From the analytic signal calculated above, phase (ϕ_*t*_) is defined as the inverse tangent of the ratio between the imaginary and real values at each time point

$$\begin{array}{l} \phi_{t}={tan}^{-1}\left(H\left(X_{t}\right)∕X_{t}\right) \end{array}$$

Phase values are expressed in radians (or degrees), and capture the angular position (counter clockwise rotation), of the complex valued coordinate, assuming the values of [−π < θ ≤ π] (see Figure 1). By obtaining the instantaneous phase signal (7–10 Hz alpha band in this example) from two regions, one can test for the consistency of their relative phase position over time (i.e., their phase synchrony):

$$\begin{array}{l} C\phi =\frac{1}{n}|\sum_{t=1}^{n}e^{i\left(△\phi_{t}\right)}| \end{array}$$

Here, the estimated phase consistency *Cϕ* between two regions (e.g., regions X and Y) is the absolute circular mean value of phase vectors reflecting the angular phase difference between regions X and Y at each time point (△ϕ_*t*_ = ϕ*X*_*t*_ − ϕ*Y*_*t*_). As each phase vector has unit length, *Cϕ* will range from [0 to 1], where 1 is perfect phase consistency. This estimate has been described as the phase locking value by Lachaux et al. (1999). A number of similar approaches exist for estimating phase correlation, with a varying nomenclature used by different authors (Cohen and Gulbinaite, 2014). However, in most cases the temporal consistency of phase signals is estimated via circular statistics. Importantly, the estimation of phase consistency requires careful consideration of type I errors, which may arise due to low statistical power (e.g., limited sample size) or contaminated measurements (e.g., volume conduction). A number of methodological improvements have been suggested to address these concerns (e.g., Stam et al., 2007; Vinck et al., 2010, 2011). While the example here focuses on the phase consistency of an alpha band signal between two regions, coupling may also occur at different frequencies between regions (e.g., 10 and 20 Hz; Tass et al., 1998).

### Summary of invasive findings

Invasive cortical recordings in humans and non-human primates provide direct insight into spontaneous neural activity at the micro-, meso-, and macro-scopic scale. Using multisite recordings, correlations of electrophysiological activity between distant brain regions can be explored, and in many cases compared directly to rsfMRI data. Using the analytical techniques described in the previous section, several investigators have sought to identify robust correlates of spontaneous covariation in cortical activity.

Obtaining simultaneous intracranial electrophysiology and fMRI data from the human brain poses a number of technical challenges such as interference and induction currents caused by MRI (Carmichael et al., 2012). As a consequence, most human studies have instead focused on comparing non-simultaneous electrophysiological and fMRI measurements within subjects who are in a common task or state. For example, Mukamel et al. (2005) recorded single units and local field potentials (LFPs) from auditory cortex of human subjects who were viewing a movie, and compared electrophysiological responses against BOLD signals recorded from auditory cortex of a separate group of subjects watching the same movie. The authors found that slow changes in spike rate and the amplitude of high-frequency (40–130 Hz) LFPs were both positively correlated with the BOLD signal, while the amplitude of low frequencies in the 5–15 Hz range were anticorrelated with the BOLD signal. These findings are broadly consistent with reports based on simultaneous BOLD-LFP recordings in animals (Logothetis et al., 2001; Niessing et al., 2005).

Knowledge of the electrophysiological correlates of BOLD activity at a single brain location does not necessarily reveal the electrophysiological processes underlying the spatially distributed functional networks observed using fMRI. As one of the first invasive electrophysiological investigations of the neural bases of rsfMRI networks in the human brain, He et al. (2008a) collected rsfMRI data in drug-resistant epilepsy patients undergoing pre-surgical evaluation using intracranial electrocorticogram (ECoG) electrodes (the main clinical setting for human intracranial studies). Resting-state fMRI data were collected either before the implantation of the ECoG electrodes or after the electrodes were removed and the epileptogenic zone resected. By co-registering the anatomical MRI with the computer-aided tomography (CT) scan, which documented the electrode locations, the authors spatially co-registered fMRI and ECoG data in each patient (an approach common to most studies reviewed here). In order to identify the neurophysiological correlates of rsfMRI networks, the authors decomposed the ECoG signal into different frequency bands, and evaluated both the raw signal and the band-limited amplitude at different frequencies. In addition, the authors compared the correlation of ECoG signals obtained during normal wakefulness, slow-wave sleep (SWS), and rapid-eye-movement (REM) sleep with rsfMRI signals.

Looking across states of arousal and across frequency bands, He et al. (2008a) observed that the ECoG signal in the low-frequency range—i.e., the delta (1–5 Hz) and sub-delta (<1 Hz) bands that constitute the so-called slow cortical potentials (SCPs), provided the best correlate of the networks defined by rsfMRI signals. This correspondence persisted regardless of whether the ECoG data were collected during wakefulness, SWS or REM sleep. The band-limited amplitude of the gamma frequency (50–100 Hz) range also demonstrated good correspondence with the fMRI resting-state signals, but this relationship was weaker than the SCPs, and it was abolished during SWS. These results revealed the SCP as a theretofore unknown correlate of the spontaneous fMRI signal. Based on these findings and previous research on the physiology of SCP, the authors proposed that the SCP may also be a good correlate of the fMRI signal more generally (He and Raichle, 2009). This prediction has been borne out by two recent animal studies employing simultaneous fMRI and DC-coupled LFP recordings (Kahn et al., 2013; Pan et al., 2013).

The findings of He et al. (2008a) identified two temporally different correlates of rsfMRI, the SCP and the (much faster) gamma band. How might these different time scales of cortical dynamics be reconciled? Some insight into this question was provided by Nir et al. (2008). In a similar investigation, Nir et al. (2008) recorded spontaneous activity invasively from both hemispheres of the human cortex and studied long-range correlation between them. These authors focused their analyses on unit spiking activity and band-limited LFP power (BLP) changes, and, innovatively, the modulation of these signals at fast (>1 Hz), medium (0.1–1 Hz), and slow (<0.1 Hz) time scales. As noted above in Figure 2, the amplitude/envelope of LFP BLP has unique spectral properties, which can be low-pass filtered to study slow fluctuations. In performing this analysis, the authors were able to consider how rapid neural dynamics, like spiking or gamma band activity, are modulated at slow time scales similar to fMRI BOLD dynamics.

Nir et al. (2008) reported that across their measures, the best correlate of long-range network activity was the slow (<0.1 Hz) modulation of spiking and gamma band (40–100 Hz) activity. For example, spatial selectivity and high correlation was found between bilateral auditory cortices for slow modulations of gamma band activity. These findings provide a link to the observations of He et al. (2008a) by indicating that gamma band correlations may be subject to slow modulations (like the SCP) that are shared between distant regions (He and Raichle, 2009). Importantly, these long-range slow modulation gamma band correlations differ from those alternatively proposed to be coherent between functional regions (Fries, 2005; Engel et al., 2013). Similar to He et al. (2008b), Nir et al. (2008) observed that long range correlations in slow modulations of gamma BLP were preserved across waking and sleep states. However, one limitation in linking the findings of Nir et al. (2008) to rsfMRI data is the lack of direct comparison to rsfMRI from the same subjects.

More recent investigations have helped address this question by applying similar data analyses to Nir et al. (2007) in subjects for which both resting-state ECoG and fMRI data were available. Keller et al. (2013) studied the spatial correlations observed with resting ECoG recordings from the lateral cortical surface (covering frontal, parietal, and temporal lobes) for fast (1–10 Hz) or slow (0.1–1 Hz) modulations of high gamma-range activity (50–150 Hz). These authors report that slow modulations of high gamma power provided the strongest inter-regional correlations, and the strongest similarity to rsfMRI interregional correlations assessed within the same subjects. Keller et al. (2013) also report that this similarity extends to anti-correlations (discussed below) observed between regions, although the BOLD-ECoG correspondence was substantially weaker for inter-regional anti-correlations. While these findings provide support for the identification of overlapping resting-state functional networks with electrophysiology and fMRI techniques, it is important to also directly confirm that regions showing putative connectivity do share functional responses under specific task conditions.

Addressing this question directly, Foster et al. (2015) recently quantified the electrophysiological correlates of spontaneous and task-based fMRI correlations in human parietal cortex. Specifically, these authors focused on simultaneous ECoG recordings from the medial and lateral parietal surface during an explicit task, as well as rest and sleep states. Across these states, Foster et al. (2015) quantified correlations across the parietal lobe of the amplitude of high-frequency activity (70–180 Hz). During task conditions that required autobiographical retrieval, the authors observed strong trial-wise correlations between the medial retrosplenial/posterior cingulate (RSC/PCC) region and the lateral angular gyrus (AG). These anatomical regions are key nodes of the default network, which displays reliable fMRI activation during episodic memory retrieval (Wagner et al., 2005; Rugg and Vilberg, 2013). During resting and sleeping states, the authors further quantified the correlation between parietal regions in slow (<1 Hz) fluctuations of spontaneous high frequency amplitude (70–180 Hz), analogous to the approach employed by Nir et al. (2008) and (Keller et al., 2013), and found correlation patterns that largely resembled those observed during the retrieval task. Next, Foster et al. (2015) compared ECoG resting-state connectivity patterns (correlation matrices) with rsfMRI data acquired for each subject, showing significant positive correlations between both modalities. Together, these findings support the view that slow time scale electrophysiological activity can exhibit strong correspondence with rsfMRI connectivity patterns, and that these slow fluctuations include the modulation of local high-frequency dynamics. In addition, these data also support the observation that spontaneous activity captures intrinsic properties of functional networks across behavioral states (e.g., task, rest, and sleep; see also Ramot et al., 2013). However, future work is required to better understand the cellular and circuit-level mechanisms driving these inter-regional correlations. One avenue for investigation toward this aim is the use of single cell recordings in non-human primates combined with fMRI imaging.

Cortical resting-state activity has been evaluated at the level of single neurons using simultaneous fMRI and intracortical neurophysiological recordings, typically in anesthetized macaque monkeys. In one study focused on the primary visual cortex (V1), macaques were either exposed to a uniform gray field or kept in complete darkness (Shmuel and Leopold, 2008). Under these conditions, the authors found that slow fluctuations in the BOLD signal were correlated with local neuronal activity following a (~6 s) time lag that roughly resembled the hemodynamic response function (i.e., the time it takes vascular tissue to respond to local changes in metabolic demand). Activity in the gamma frequency band (24–90 Hz) as well as the local population spiking showed high correlations with the BOLD response. When the authors correlated the time-varying amplitude of these measures of local neurophysiological activity with the fMRI time-courses of voxels across the entire visual cortex, they found widespread co-activation of visual cortical areas across both hemispheres. These results suggest that, at least for the visual system, rsfMRI-based networks might be linked to synchronization of slow fluctuations in spiking activity across anatomically distinct, but functionally related brain areas (see Logothetis et al., 2009 for caveats). Although previous work suggests spontaneous correlations are intrinsically preserved across different states of awareness (Liu et al., 2015), the effect of anesthetic compounds likely influences the covariation structure of spontaneous dynamics.

More recently, simultaneous rsfMRI and neurophysiological recordings have been performed in alert non-human primates resting in a dark environment (Schölvinck et al., 2010). The authors focused on widespread, positive correlations of fMRI signals that are frequently excluded from resting-state functional connectivity analyses to correct for motion artifacts. Strikingly, the authors observed that the gamma-range activity (40–80 Hz) measured at a single cortical site was well-correlated with this global rsfMRI signal (Schölvinck et al., 2010). More specifically, the neuronal signal recorded at a single cortical site accounted for up to 10% of the global BOLD signal variance, suggesting a neuronal origin for these brain-wide BOLD correlations at rest. Taken together, the findings from these two studies suggest that fMRI fluctuations measured during the resting-state are closely linked to neural activity.

The observation that spontaneous fluctuations in gamma range activity correlates with rsfMRI signals in non-human primates is consistent with studies observing a tight coupling between local BOLD activation and gamma band activity recorded from the same brain area in humans. As discussed above, human ECoG studies have reported that inter-areal correlations in gamma power are linked to BOLD correlations between these areas (He et al., 2008a; Nir et al., 2008; Keller et al., 2013; Ko et al., 2013; Foster et al., 2015). An important technical qualification is that few studies use the same frequency range to define gamma activity, or the same terminology to describe frequencies between 40 and 200 Hz (e.g., gamma, high-gamma, broadband). Importantly, despite these differences, growing evidence suggests that changes in high frequency activity >40 Hz recorded from the human cortex has a broadband spectral representation, and the different subsampling of this frequency range will approximately track the same temporal process (Miller et al., 2014b). Importantly, high-frequency spectral changes also track event-related cortical deactivations (Shmuel et al., 2006; Lachaux et al., 2008; Ramot et al., 2012), particularly within cortical regions comprising the default network, in both humans (Miller et al., 2009; Jerbi et al., 2010; Dastjerdi et al., 2011; Ossandón et al., 2011; Foster et al., 2012) and non-human primates (Hayden et al., 2009). While debate still surrounds the appropriate biophysical interpretation and statistical treatment of this spectral range (Brunet et al., 2014; Hermes et al., 2015a,b; Podvalny et al., 2015; Ray and Maunsell, 2015; Gao, 2016), growing evidence suggests there are distinct neural processes that generate spectrally isolated (oscillatory) gamma and spectrally broad (asynchronous) high-frequency components that appear in the >40 Hz range (Buzsáki et al., 2012; Lachaux et al., 2012; Ray and Maunsell, 2015).

In addition to correlated changes in the “gamma” range, several studies reviewed above also showed significant, although more spatially diffuse, correlations in the power of lower frequency oscillations between brain areas. Electrodes on the cortical surface used for ECoG studies most likely reflect activity of neurons in the supra-granular cortical layers (Kajikawa and Schroeder, 2011; Fukushima et al., 2012). In contrast, studies in non-human primates (e.g., Katzner et al., 2009) measure activity in superficial or deep cortical layers, depending on electrode depth. There is growing evidence that different cortical layers show different spectral profiles, for example, higher gamma power in superficial layers, and more prominent low frequency activity in the deep layers (Maier et al., 2010; Buffalo et al., 2011; Xing et al., 2012; Smith et al., 2013; Godlove et al., 2014; Ninomiya et al., 2015; but see Lakatos et al., 2005, 2008). Low frequency oscillations (e.g., in the theta and alpha range) are generated by certain neuronal populations in the deep layers of cortex (Lopes Da Silva and Storm Van Leeuwen, 1977; Lopes Da Silva, 1991; Silva et al., 1991; Bollimunta et al., 2008, 2011; Sun and Dan, 2009) as well as in the thalamus (Hughes and Crunelli, 2005; Lörincz et al., 2008), which projects to a subset of cortical layers. Low-frequency contributions to the BOLD signal thus may be more difficult to detect in studies that do not independently sample form the different cortical layers.

To measure how much low-frequency activity contributes to rsfMRI correlations, Wang et al. (2012) investigated a higher-order thalamocortical network. To do so, Wang et al. (2012) simultaneously recorded neuronal activity from four distributed sites in visual cortex (V4), inferior temporal cortex (TEO), posterior parietal cortex (LIP), and the thalamus (pulvinar) of macaque monkeys during the resting-state. The authors compared this electrophysiological network activity to correlations in rsfMRI BOLD signals acquired under the same conditions. Analyses of slow-varying changes in LFP BLP, as well as the phase of LFPs, showed that low frequency activity (<20 Hz) predicted resting-state BOLD correlations between all pairs of network sites. Specifically, on a fast time-scale, Wang et al. (2012) showed the highest coherence between neural activity from distant sites to be at theta/alpha frequencies. Similar to the analyses described above for human ECoG data, the authors also filtered time series of theta/alpha power into timescales matching the slow fluctuations of the BOLD signal (0.01–0.1 Hz), and found that the highest correlations between these band limited power time series also occurred at theta/alpha frequencies. Non-invasive work in human subjects provides further support for a broad range of frequencies, in addition to gamma, whose inter-regional coupling is associated with the correlations observed in rsfMRI (Laufs et al., 2003; Mantini et al., 2007; de Pasquale et al., 2010; Brookes et al., 2011; Hipp et al., 2012; Marzetti et al., 2013; Cabral et al., 2014; Hipp and Siegel, 2015).

The prominent role of low frequency oscillations can be reconciled with evidence of gamma contributions to BOLD signals by considering cross-frequency coupling mechanisms (i.e., modulation of higher frequency components by lower frequencies). Wang et al. (2012) investigated this possibility by first calculating the phase coherence between the phase of low frequency (alpha-range 8–13 Hz) oscillations between recordings sites. Secondly, the authors calculated how the phase of this low frequency oscillation modulated local high frequency gamma activity within each recording site (Cohen, 2008). This cross-frequency coupling analysis suggested that the phase of low frequency oscillations was coherent between sites, and that it locally modulated the amplitude of gamma oscillations within sites (Wang et al., 2012). This suggests that the long-range correlation of gamma power between brain areas may be driven by lower frequency (e.g., alpha) coherence between regions, which locally modulate ongoing gamma power.

The findings reviewed above beg the question of how neurons in different brain areas synchronize. There are extensive and reciprocal anatomical connections between the thalamus and cortex (Sherman and Guillery, 2006; Jones, 2007), supporting the notion that thalamocortical interactions are important for generating synchronous oscillatory activity across the brain. In addition to the first-order sensory relay nuclei in the thalamus (e.g., lateral geniculate, ventral posterior nuclei), which receive input from cortical layer 6 only, there are also higher-order thalamic nuclei (e.g., pulvinar, mediodorsal nucleus), which constitute the major volume of the thalamus and receive input from cortical layers 5 and 6. The higher-order thalamus respectively projects to cortical layer 4 and more superficial layers, providing indirect pathways between cortical areas, which are well-positioned to influence functional connectivity across the cortex (Shipp, 2003; Jones, 2009; Saalmann, 2014). As mentioned above, the thalamus generates low frequency oscillations, e.g., alpha (Hughes et al., 2004; Hughes and Crunelli, 2005; Lörincz et al., 2008), and thalamic lesions affect low frequency oscillations in the cortex (Ohmoto et al., 1978). Other studies have shown that the pulvinar regulates the degree of alpha- and low-beta synchrony between visual cortical areas based on behavioral demands (Saalmann et al., 2012). Consistent with the idea of a closely coupled thalamocortical system, human and monkey studies have reported robust resting-state networks incorporating the thalamus (Zhang et al., 2008; Wang et al., 2012). This suggests that the thalamus may be a vital node for supporting resting-state networks.

In addition to low frequency subcortically mediated synchrony, long-range coordination may also be achieved through cortico-cortical high-frequency synchrony (Fries, 2015; Bastos et al., 2015b). A number of investigations have specifically highlighted the role of gamma range (40–80 Hz) synchrony in dynamic functional brain networks (e.g., Bastos et al., 2015a), which provides an alternative mechanism for coordinating intrinsic network patterns (Engel et al., 2013). While extant data has chiefly focused on the role of gamma synchrony during task conditions, it is of future interest to consider the spontaneous organization of gamma synchrony patterns, and to see how these rapid long-range dynamics may relate to the slower time scales of modulation described above (Bressler and Richter, 2015; Womelsdorf and Everling, 2015).

## Correlated spontaneous activity across different scales of brain organization

Invasive and non-invasive electrophysiological recordings from both humans and non-human primates suggest a number of, possibly related, neuronal correlates of spontaneous hemodynamic activity observed with rsfMRI. While comprehensive electrocortical mapping is challenging, focused efforts on specific networks suggest a strong overlap between the macro-scale areal parcellation of functional cortical regions through covariance structures in spontaneous activity using either modality (He et al., 2008b; Wang et al., 2012; Keller et al., 2013; Foster et al., 2015). Given the striking correspondence between spontaneous and task-based parcellations of large-scale functional brain networks, it is of interest to consider the extent to which spontaneous activity may also reveal meso- and possibly micro-scale organization within brain regions. For example, sensory cortices display robust long-range correlations, such as spontaneous correlations between primary auditory or visual areas (Nir et al., 2008; Yeo et al., 2011). However, within these areas there is well-documented topological organization (i.e., retinotopy or tonotopy). Does spontaneous activity, when measured with sufficient spatial resolution, conform to this within-region topology?

Recent electrophysiological investigations suggest that spontaneous cortical activity on the mesoscopic scale also captures local, within-region functional organization. A useful bridging example between inter-areal and intra-areal organization, is provided by Fukushima et al. (2012). Fukushima et al. (2012) utilized micro-ECoG recordings from the plane of the superior temporal sulcus in macaque monkeys to study evoked and spontaneous electrocortical activity within tonotopically organized auditory cortex. First, Fukushima et al. (2012) established the ability to identify tonotopic cortical organization using task-evoked responses. Importantly, the authors focused on changes in the high-gamma range (50–150 Hz), analogous to the studies reviewed above. Using these task-based tonotopic maps as functional templates; the authors studied the spatial organization of spontaneous activity, again focusing on changes in high-gamma amplitude. Following this approach, the authors found high similarity between the spatial configuration of spontaneous and task-based activity patterns, such that spontaneous data could be used to estimate the predominant tonotopic organizational structure within auditory cortex.

Analogous to the human ECoG work reviewed above, the findings of Fukushima et al. (2012) suggest a pivotal role for spontaneous high-frequency activity (high-gamma, 50–150 Hz). Importantly, these authors focused on the direct changes in high-frequency amplitude, rather than slower fluctuations of high-frequency amplitude/power (Nir et al., 2008; Wang et al., 2012; Keller et al., 2013; Foster et al., 2015). This difference in time-scale likely influences efforts to study different spatial scales of covariation, as low-pass filtering of the high-frequency amplitudes removes more localized activity, and instead favors slower, more diffusely correlated modulation of distant regions. Under this view, there are likely heterogeneous time scales of modulation present within spontaneous activity patterns, which relate to the different spatial and temporal constraints of local and distal network organization. This “nesting” of temporal dynamics across spatial scales is an important area for future investigation, however, there is some evidence suggesting infra-slow EEG activity appears to modulate higher frequency activity, and also correlate with rsfMRI networks (Monto et al., 2008; Palva and Palva, 2012; Hiltunen et al., 2014).

While both human ECoG and micro-ECoG measurements in macaques provide clear evidence for strong correspondence between local spontaneous and task-related patterns on the level of neuronal population activity, such correspondence has also been documented at the level of single neurons. In a series of seminal investigations, Arieli et al. reported a striking correspondence between the spatial organization of spontaneous and task-evoked activity by triggering optical imaging data of the primary visual cortex in the cat to the spiking activity of isolated neurons (Arieli et al., 1995, 1996), Specifically, they found that “snapshots” of local cortical activation at the time of elicited spikes were nearly indistinguishable between spontaneous and task-related responses. This correspondence between ongoing spontaneous activity and evoked responses, has been extended and replicated in visual (Tsodyks et al., 1999; Kenet et al., 2003) as well as in somatosensory cortices (Wang et al., 2013). These studies, combined, suggest that the influence of spontaneous activity on task-evoked responses is a critical factor for the variability of neural responses across repeated stimulus presentations (see discussion below). Interestingly, these studies show that the similarity between spontaneous activity patterns and task-evoked functional organization progressively increases over early development, indicating shared sensitivity to anatomical and functional sculpting of the underlying neural tissue (Fiser et al., 2004; Berkes et al., 2011). Therefore, in the developed brain, spontaneous activity reflects basic organizational properties of local neural populations, and at the same time influences the response properties of that population. This influence exerted by spontaneous activity can be a non-trivial factor on the computational properties of neural populations. Indeed a large body of work has focused on studying the influence of “noise-correlations” on population coding (Averbeck et al., 2006).

The fact that spontaneous and task-elicited dynamics exhibit common patterns of covariance would not be especially surprising if one were to consider both as being determined by a common underlying anatomical connectivity. However, it can be more useful to think of monosynaptic anatomy as shaping and constraining, but not determining, the covariation in population activity (Honey et al., 2009). A clear example of this principle is provided in the visual cortex, where task-driven and spontaneous BOLD fluctuations are generally correlated among regions and subregions that are known to be anatomically connected (Nir et al., 2006; Yeo et al., 2011). At the same time, however, there are departures from a direct correspondence: inter-regional correlations are stronger among subpopulations that represent the same visual eccentricity, more so than sites that represent the same polar angle (Yeo et al., 2011; Arcaro et al., 2015). For example, the functional connectivity between circuits in human V2 and V3 reflect much more than the presumed monosynaptic reciprocal connections between neurons with common receptive fields. Similarly, as noted by Genç et al. (2015), the fact that foveal and peripheral sites within V1 are correlated despite lacking monosynaptic connections, again argues against monosynaptic links as the only driver of covariance, even within brain areas. Thus, anatomy shapes covariance, but does not constrain it to immediate first order interactions, allowing local and global network covariance. Indeed, for slow hemodynamic signals such as BOLD fMRI, there is ample room for covariance to be shaped by multi-synaptic causal paths, by common input to causally unconnected sites (e.g., from subcortical modulators), and, finally, as we discuss elsewhere in this review, by multi-scale emergent dynamical processes in the primate brain.

### Neural noise correlations

One striking property of neuronal responses is the degree of variability in spiking activity across repeated presentations of a sensory stimulus. This trial-by-trial response variability is shared across specific members of the neuronal population (Cohen and Kohn, 2011). The degree of shared variability (around the mean response) across trials between neurons is typically referred to as noise correlation (Averbeck et al., 2006; Cohen and Kohn, 2011). The somewhat pejorative use of the term *noise* to describe these correlations has an historical explanation, as inter-regional and inter-neuronal correlations were originally studied from the perspective of the information capacity of population codes. In the language of Shannon's theory of information, a high level of independent spiking across a population of neurons carries a high amount of information (Zohary et al., 1994). Conversely, as the level of noise correlation increases across a population, the informational content that could theoretically be decoded at the next stage of processing (which receives this population activity readout as sole input) is reduced as similarly firing neurons become redundant (Averbeck et al., 2006). Experimental and computational work has extended this simplistic view to a more nuanced account of how noise correlations relate to the functional repertoire of neuronal populations and their relative size (Kohn et al., 2009). Importantly, it has become clear that the degree of noise correlations during experimental stimulation provides insight into the shared interactions and inputs between members of local populations (Ringach, 2009). In addition, more recent evidence suggests that these properties are present outside of task performance (Okun et al., 2015), and show important sensitivities to the context of behavioral states (Ecker et al., 2014; Schölvinck et al., 2015).

Above, we reviewed work that identified *topographical* organization in patterns of spontaneous activity at the surface of sensory cortex. It is of great interest whether or not spontaneous activity covariation may inform functional organization of non-sensory, associative cortical areas. A key challenge here is the uncertainty that functional organization conforms to cortical topography. Consideration of noise correlations has shown to be useful in this regard. For example, Kiani et al. (2015) recently addressed these questions directly by studying the statistical grouping of neurons in frontal cortex of the macaque. Kiani et al. (2015) studied the correlation of spiking activity between isolated pairs of neurons recorded across a high-density electrode array (Utah array) implanted in the prearcuate gyrus. Using unsupervised data clustering methods, the authors found that neuronal spiking during a visual task was functionally clustered within the prearcuate gyrus. Functional clustering was defined by the similarity of spiking responses across units. There were approximately two clusters of neurons showing dissociable response properties, which were also spatially dissociated across the recording array over the prearcuate gyrus. Importantly, the authors observe this clustering structure across all periods of the task, collectively (i.e., entire task) and in isolation (i.e., different trial periods).

Neuronal response similarity across different task periods supports the view that the correlations driving clustering were due to shared “noise correlations” between neurons. To quantitatively confirm this inference, Kiani et al. (2015) analyzed the data focused only on the residual responses across trials, by subtracting the mean normalized activity during the task. This approach follows analytical approaches outlined above, where response variability across trials is correlated between neurons to estimate their shared “noise.” The authors emphasized the analytical similarities between their work and common methods employed for large-scale parcellation of functional regions using rsfMRI. Importantly, the findings of Kiani et al. (2015) suggest that spontaneous activity is informative for functional grouping at the level of single neurons within higher order associative cortices for which topographical organizational principles are challenging to elucidate. It is of importance to understand how these local observations can be linked to the large-scale parcellations/networks observed with fMRI and electrophysiology. However, one promising observation was that similar parcellation across the recording array could be achieved using low frequency activity (Kiani et al., 2015).

## Dynamic structure of correlated spontaneous activity

As reviewed above, spontaneous neural activity at multiple scales of cortical organization contains spatial patterns of covariant activity that reflect functional and anatomic constraints. Given this correspondence, and the reliability of topological cortical organization, it is worth considering whether the correlated time courses and ongoing dynamics of spontaneous activity also reflect other properties of neuronal networks. Following the intuition promoted here, spontaneous activity is determined, or at the very least constrained, by anatomical and physiological properties. Therefore, it is possible that spontaneous activity will conform to certain types of dynamics permissible by neural tissue.

As noted above, several investigations have reported topographically organized spontaneous activity patterns that recapitulate sensory maps across the cortical surface. A more striking question is whether spontaneous neural activity at the spiking/ensemble level, e.g., within an orientation column of visual cortex, displays dynamical motifs of temporal behavior. This question has been addressed in a series of important studies by Yuste and colleagues, who utilized calcium-imaging methods to record and compare ensemble-spiking activity during spontaneous and evoked states (Cossart et al., 2003; MacLean et al., 2005; Miller et al., 2014a; Carrillo-Reid et al., 2015).

A foundational question for quantifying structures in spontaneous ensemble activity is whether ongoing spiking displays repeating patterns of ensemble activity. Using two-photon calcium imaging, Cossart et al. (2003) studied the patterns of ensemble spiking in slice preparations of rodent visual cortex. During periods of spontaneous upstates, the authors observed repeating stereotyped ensemble activity, which reflected a “core” of regularly activated neurons. Given these stereotyped responses during spontaneous states, it is of interest to compare how the responses of single neurons relate to functional activation of the ensemble. To study this question, MacLean et al. (2005) employed two-photon calcium imaging in thalamocortical slices that covered somatosensory cortex. Using this preparation, the authors were able to quantify ensemble-spiking activity triggered by both spontaneous activity and thalamic stimulation. Following previous observations, MacLean et al. (2005) identified consistent ensemble patterns of activity during spontaneous upstates. Stereotyped patterns of ensemble activity in somatosensory cortex were also observed during activation via electrical stimulation of the ventral thalamus, which also reflected a local depolarizing upstate. Strikingly, MacLean et al. (2005) observed no discernable difference between the ensemble patterns identified during spontaneous or thalamic triggered upstates, which was confirmed electrophysiologically via intracellular recordings of target cells. This similarity between conditions more generally supports the conclusion that spontaneous dynamics were not driven by thalamic inputs, as tested with this preparation. These observations lead the authors to speculate that thalamic feed-forward excitation works to release dynamic “trajectories” of activity that are intrinsic to cortex (MacLean et al., 2005). Again, this conclusion suggests that spontaneous dynamics follow patterns of network activity that conform to the predominant causal pathways of circuit interaction. Such claims, however, are limited by the fact that *in vitro* sampling of network activity lacks many aspects of neural activity *in vivo*, such as cortico-cortical feedback and neuromodulation from nuclei in the brain stem.

More recently, Miller et al. (2014a) addressed this technical limitation by studying ensemble population activity using two-photon calcium imaging of visual cortex in awake rodents during spontaneous and visually stimulated conditions. These experimental conditions provide important challenges for previous observations, as the *in vivo* setting presents a far richer neural context of causal influence, and therefore the possibility for divergence of ensemble activity patterns between spontaneous and task evoked states. Comparing spontaneous periods to the presentation of visual gratings and naturalistic movies, Miller et al. (2014a) observed that the same ensembles of neurons were coactivated across conditions. This experimental preparation did not include electrophysiological recordings, and so explicit temporal similarity of spiking activity was not captured. However, consistent with the slice work reviewed above, these observations suggest that coactivation of a “core” ensemble of neurons is key to the intrinsic functional state of a cortical region. Indeed, Miller et al. (2014a) speculate that evoked functional responses sample from a “lexicon” of intrinsic functional ensemble configurations that are transiently expressed in spontaneous activity, in the absence of sensory events. Importantly, while this work focused on the identification of spatial ensembles, recent analysis of these data, focused on time-resolved extraction of ensemble similarities, further supports the view that canonical sequences of ensemble engagement during visual stimulation also occur during spontaneous states (Carrillo-Reid et al., 2015). Endogenous patterns of neural activity therefore provide a strong prediction of the possibility space of functional states during stimulation, even under conditions where animals are naïve to the experimental stimuli—excluding related phenomena such as preplay or replay of firing sequences (Carrillo-Reid et al., 2015).

The hypothesis that neuronal task and spontaneous dynamics explore a common state space requires further support from studies with high-temporal resolution. Using tetrode recordings from rodent primary auditory cortex, Luczak et al. (2009) studied similarities of spontaneous and sensory-evoked population spiking activity. The authors first characterized the responses of neurons to tones and naturalistic stimuli. Although neurons differed in their stimulus-triggered spiking responses, each isolated neuron displayed stereotyped consistency of spiking activity for tones of differing frequency and naturalistic stimuli. Next, Luczak et al. (2009) studied the spiking activity of the same neurons during spontaneous upstate periods. Strikingly, the authors observed a strong correspondence between the stereotyped responses of a given neuron across tones, naturalistic sounds and spontaneous upstates. Importantly, the authors also confirmed that this similarity between evoked and spontaneous events is observed regardless of whether the animals are awake or anesthetized.

Luczak et al. (2009) interpret the similarity of spiking sequences across states to reflect a set of constraints imposed on the population that greatly limit the repertoire of neural responses. To further explore this conjecture, the authors studied population spiking as multi-dimensional vectors of time-integrated firing rates for each recorded neuron. In considering the simplest case of two neurons, Luczak et al. (2009) observed a strong overlap in responses of the two neurons during evoked auditory stimuli and spontaneous events, whereby spontaneous events occupied a large, structured 2-dimensional space that included the evoked events. Importantly, spontaneous activity did not occupy all of the response space uniformly, and was geometrically different from the space occupied by randomly shuffled data. This observation was extended to a multi-dimensional space across multiple neurons. When visualized in a 2-dimensional projection, the population vectors of spiking responses for different stimuli all occupied small clusters within the space formed by spontaneous activity, which itself was topologically distinct from randomly shuffled data. From this observation, the authors inferred that spontaneous activity explores and therefore conveys the boundary conditions of population dynamics, which form the shared constraints imposed on evoked responses. Given these limits, the authors suggest that functional responses of a local circuit population are constituted by a limited set, or “vocabulary,” of activity patterns, from within a neural response space outlined by spontaneous neural dynamics.

What might be the sources of these shared constraints? A classical interpretation is to view local circuits as physio-anatomic ensembles sculpted through maturation by shared statistical history of activation (Hebb, 1949; Fuster and Bressler, 2012). Consistent with the general intuition advocated in this review, the Hebbian formation of local and global cell assemblies suggests that these spatio-temporal attributes, or constraints, will be expressed in ongoing spontaneous activity. Partial empirical support for this view, where ontogenetic and environmental factors sculpt circuit formation, is derived from work reviewed earlier, which shows that the similarity of topographic patterns in visual cortex during spontaneous and evoked states increases across maturation (Fiser et al., 2004; Berkes et al., 2011). As discussed below, acute changes in neural dynamics, such as ensemble firing sequences, can occur via learning. These findings, combined, lead to interesting predictions about the factors influencing the similarity between spontaneous and evoked activity patterns, and the possibility of intervening in circuit organization to compare the reconfiguration of population dynamics during spontaneous and stimulated events.

### Preplay/replay spike sequences

The electrophysiological and optical imaging data reviewed above suggest that neural populations display patterns of spontaneous activity that relate closely to evoked responses. This relationship suggests that using appropriate multivariate methods, functional response patterns of neuronal populations can be predicted from the statistical attributes observed during spontaneous non-task states (Luczak et al., 2009; Okun et al., 2015). How might these observations from sensory cortical regions relate to similar phenomena of spike sequence preplay and replay in the hippocampus?

Place cells within the hippocampal subfields display spatial receptive fields, which modulate their firing rate as the animal traverses a preferred topological location (i.e., a place field). Recording multiple place cells with overlapping receptive fields will therefore display a progressive sequence of spiking activity as the animal moves through each successive place field (Lisman and Redish, 2009). Strikingly, it has been repeatedly observed that both prior to, and after, a learned locomotor trajectory is taken, the unique firing sequence across mapped placed cells is repeated (Diba and Buzsáki, 2007; Pastalkova et al., 2008). For both preplay (repeated spike sequence prior to locomotion) and replay (repeated spike sequence post locomotion), firing patterns are triggered by transient high-frequency activity called “ripples” (Buzsáki, 2015). Interestingly, these spiking sequences can run in the forward direction, matching locomotion sequence, or backward direction (Diba and Buzsáki, 2007), with the proportion of forward/backward sequences modulated by behavioral state (Wikenheiser and Redish, 2013). More recent experimental work has shown that preplay sequences not only reflect learned sequences, but also express sequences reflecting novel trajectories of navigation and planned behavior (Wikenheiser and Redish, 2015; but see Silva et al., 2015). Given that these events happen outside of explicit behavioral events, to what extent do they mirror the relationship between spontaneous and evoked activity patterns?

Preplay and replay spike sequences differ in their relationship to task performance. While these sequences do occur during passive states, these states occur within a specific temporal context of task performance. In this fashion, they likely reflect a clear behavioral purpose related to the consolidation and learning of behavior, as well as to support planned action and decision-making. Making similar inferences about spontaneous data is difficult given the lack of explicit behavioral controls. However, preplay/replay phenomena in the hippocampus may be thought of as a well-documented case of how ensemble dynamics are strongly constrained by anatomical and biophysical factors, whereby local excitable events (e.g., ripples) trigger a limited set of spiking activity. In the case of preplay/replay, recent learning, and therefore activation of specific firing sequences, will bias the population to an even smaller response space when subject to excitatory drive. These general predictions suggest spontaneous activity may be biased by recent modulations of ensemble physiology, following a Hebbian rule, and that specific sequences can be released through acute learning and controlled excitation (Luczak et al., 2009; Lewis et al., 2016). Consistent with this prediction, Xu et al. (2012) have previously shown that V1 ensemble firing sequences to a learned trajectory of a visual target can be reproduced by presenting a transient visual stimulus at the starting location of the sequence.

## Future directions

As reviewed above, spontaneous neural dynamics at multiple scales conform to basic anatomical and physiological constraints. Given this relationship, quantitative consideration of spontaneous activity patterns can provide provisional insight into the anatomical and dynamical properties of neural networks at the micro-, meso-, and macro scale. However, these inferences need to be made cautiously, as the measurement of neural activity is often confined to a limited spatial and temporal scale, and each measurement technique is biased to detect different domains of the underlying neuronal circuit functions. In addition, correlation and other statistical treatments of spontaneous data provide uncertain descriptions of underlying causal interaction. As briefly discussed below, these challenges can be addressed through improved statistical methods and causal experimental manipulations.

### Correlation, connectivity, and causal inference

The challenges posed by the complexity of brain data are pervasive in neuroscience, and are particularly salient when investigating the neuronal basis of spontaneous brain activity across multiple scales. In many experimental settings, only restricted observations of interacting neural elements (e.g., cells/circuits/regions) can be achieved, often without the ability to causally intervene. These technical limitations highlight two important constraints of correlational methods for evaluating spontaneous brain activity.

First, incomplete measurements of neural interactions limit the inferential power of observed correlations, as hidden factors may shape covariance. Indeed, a wide-ranging set of causal interactions can explain the same correlational structure. For example, regions A and B may exhibit correlated activity because they are directly connected, but this can also arise because of an indirect connection from A to B via a third region C, or from a common feed forward source, such as when C projects to both A and B. These alternative scenarios cannot be distinguished using correlation-based data analysis alone (when C is not observed). Thus, although at an aggregate level, rsfMRI covariance tends to follow anatomical connectivity, it does present some departure from tractographic connectivity estimates (Honey et al., 2009).

Secondly, correlation quantifies an undirected non-causal relationship between two variables. For example, correlations between two brain structures, A and B, may reflect a unidirectional or bidirectional causal interaction, or alternatively reflect no causal influence between elements, but rather a shared causal input from C. In this respect, causal information is essential to elucidating the actual structure of functional network topology. Ideally, casual inference is made through carefully controlled intervention of network/element interaction.

The precision required for causal experimentation is often limited to invasive animal model systems, and some rare settings in humans (Keller et al., 2011, 2014). However, there are several analytical methods that can be employed to partially deal with the limitations of the correlational approach. To address the degeneracy of coupling models that account for neural observations, it is necessary to employ methods that leverage additional assumptions (Yatsenko et al., 2015). Going beyond simple bivariate covariance measures, available methods range from relatively simple inverse covariance models (Smith et al., 2011), auto-regressive causal models (Seth et al., 2015), Bayesian networks (Mumford and Ramsey, 2014), lagged-coordinate embedding approaches (Sugihara et al., 2012), as well as brain-specific approaches such as dynamic causal modeling (Friston et al., 2003); each trade off inferential power with added assumptions. Within many of these frameworks, assumptions of sparsity are used to mitigate the problem of false-positive connectivity that can arise from indirect influences (Bolstad et al., 2011). Put more simply, it is generally assumed that a correlation matrix of measured elements likely overestimates the underlying statistical relationships. In turn, many data driven approaches seek to reduce complexity (dimensionality) by considering each pair-wise correlation relative to all other correlation pairs to help identify redundancy (e.g., partial correlation). Such approaches reflect a blind method for correcting overestimation, where correlation (regression) estimates are penalized (regularized) with respect to the remaining observed data (Tibshirani, 1996). However, with knowledge about the system under investigation and the statistical attributes of the measurement technique, more explicit forms of penalization can be applied to correlation matrices to eliminate false positives (Yatsenko et al., 2015). Improvements in these methods are becoming increasingly important as neuroscientific data sets grow exponentially in size. Finally, given the common use of linear methods, the influence of non-stationarity must be carefully considered, particularly given the growing focus on temporally resolved analyses (Hutchison et al., 2013).

Although, analysis techniques can be used in cases where causal experiments are challenging, some progress has been made for invasively studying casual connectivity in the human brain. Using intracranial recordings (ECoG), Keller et al. (2011) measured causal interactions between recording sites using cortico-cortical evoked potentials (CCEPs). To measure CCEPs, cortex is stimulated at one cortical region (by running current between a pair of overlying electrodes) and stimulation-locked responses are measured at electrodes located at other sites on the cortical surface. Keller et al. (2011) measured resting BOLD functional connectivity in a group of six patients, and mapped CCEPs across the lateral cortical surface of the same individuals. They found that the magnitude of the CCEPs between sites was correlated with the strength of BOLD functional connectivity between those sites. This relationship between spontaneous BOLD correlations and CCEPs was specific to positive BOLD correlations; regions with anticorrelated BOLD signals (discussed below) showed unreliable CCEPs.

In a follow-up study with a larger patient sample, Keller et al. (2014) mapped the large-scale causal network composed of inter-regional CCEPs. They observed that, while short-range (<5 cm distance) causal links were often reciprocal, the reciprocity over longer distances (>5 cm) was no more than expected by chance, indicating that long-range connections are often unidirectional. Peri-rolandic circuits exhibited surprisingly widespread and powerful connections in these analyses, in contrast with their typically sparse BOLD correlations. Although causal stimulation analyses are still a developing field, findings of connection reciprocity and density constrain models of functional brain architectures. The ability of CCEPs to resolve the directionality of neural information flow in the human brain makes them crucial for understanding large-scale spontaneous neural dynamics (David et al., 2010).

### Anti-correlation?

Electrophysiological measurements will assist us in moving beyond the question of *whether* two regions are functionally connected to a description of *how* two regions are interacting. More specifically, electrophysiological data will be crucial in transitioning from static to dynamic accounts of large-scale network configuration (Hutchison et al., 2013). For example, it is crucial to determine whether the information transmission between cortical regions is primarily feed forward (i.e., *driving*) or feedback (i.e., *modulatory*) in nature. Similarly, it is important to distinguish which inter-regional interactions are effectively excitatory and which are effectively inhibitory (suppressive). It has proven difficult to resolve these questions using functional neuroimaging. The literature harbors a decade of debate over whether functional antagonism can be inferred from BOLD anti-correlation (i.e., from the fact that increased BOLD signal in one site is associated with decreased BOLD signal at another site). On the one hand, the pattern of BOLD anti-correlations has been put forward as a powerful principle of large-scale neural organization (Fox et al., 2005) with clinical implications (Kelly et al., 2008; Fox et al., 2014). On the other hand, BOLD fMRI anti-correlations may arise from artifacts of acquisition, physiology or signal processing (Chang and Glover, 2009; Murphy et al., 2009; Weissenbacher et al., 2009; Saad et al., 2012).

Electrophysiological investigations can help resolve uncertainties associated with interpreting BOLD anti-correlations. Keller et al. (2011) observed that positive BOLD correlations were consistently associated with causal CCEPs, while negative BOLD correlations were not. Subsequently, Keller et al. (2013) observed that, while most negative BOLD correlations do not correspond to negative correlations in electrophysiologically measured population activity, a subset of BOLD anti-correlations matches anti-correlations of 50–150 Hz power measured electrophysiologically. Thus, a minority of anticorrelated rsfMRI networks may reflect suppressive interaction at the population level. In addition, electrophysiological measures allow us to resolve how rsfMRI correlations vary over time, which may reveal further antagonistic relationships. For example, a link with zero aggregate correlation may in fact be due to rapid switching between antagonistic and supportive modes of interactions. Although the interpretation of BOLD anti-correlations remains difficult, it is clear that electrophysiological methods with high temporal resolution and broad fields of view will be important in resolving these and related questions. Importantly, under task conditions, human intracranial data provides strong support for dynamic anticorrelated activity. For example, Ossandón et al. (2011) reported brain-wide anti-correlation patterns in human intracranial recordings closely matching prior fMRI findings. Ossandón et al. (2011) observed these anti-correlations in broadband 60–140 Hz activity during an active visual search task. Such data provide a clear electrophysiological basis for the correlation and anti-correlation patterns typically seen with fMRI within and between the “task-positive” and “task-negative” networks.

### Pathological spontaneous activity

There is now a large literature that applies rsfMRI network analyses to different neurological and psychiatric patient populations. This literature has been extensively reviewed elsewhere (He et al., 2007; Fox and Greicius, 2010; Zhang and Raichle, 2010; Fornito et al., 2015). Importantly, while neurological disease is often typified by explicit brain pathology and injury, such explicit causes have not been as readily identified in psychiatric illness. Rather, psychiatric illness can be viewed as perturbations in the functional organization or temporal dynamics of otherwise superficially healthy neural tissue (Bassett and Bullmore, 2009). Clinically, this presents a challenge for identifying pathological network structure and dynamics as biomarkers for treatment. Initial applications of rsfMRI have provided promising results in psychiatric disorders ranging from schizophrenia (Yang et al., 2014) and autism (Gotts et al., 2013) to depression (Ressler and Mayberg, 2007). In addition, a bold, contemporary initiative seeks to elucidate the functional-anatomical markers that correlate with emotional, perceptual and cognitive symptoms common to different psychiatric disorders, and to use these neural correlates as therapeutic targets (Insel, 2012; Fox et al., 2014). As part of this endeavor, consideration of spontaneous neural dynamics can help contribute to functional brain network identification and the quantification of underlying differences in network organization across psychiatric conditions (Fornito et al., 2015). Studies focused on spontaneous dynamics may also help support alternative accounts of differences in functional network dynamics associated with psychiatric disease (e.g., Uhlhaas and Singer, 2006, 2010).

## Conclusion

Evidence from multiple scales of neural organization suggests that consideration of spontaneous neural dynamics can provide insight into the basic organization of functional neural systems. Importantly, the recent interest in rsfMRI investigation of large-scale brain networks is supported by a clear correspondence to invasively recorded neural activity in humans and non-human primates. These invasive recordings have further revealed important neural organizational principles at the mesoscopic and microscopic scales. As we gather more information about the electrophysiological basis of large-scale neural connectivity and dynamics, a major goal should be moving from descriptive to more mechanistic models of neural dynamics and network organization. This progress is a necessary step toward harnessing the power of spontaneous brain dynamics for more effective treatments of psychiatric and neurological disorders.

## Author contributions

All authors listed, have made substantial, direct and intellectual contribution to the work, and approved it for publication.

## Funding

This work is supported by a career development award from the US National Institute of Mental Health (K99MH103479) to BF; the intramural research program of the National Institutes of Health/National Institute of Neurological Disorders and Stroke for BH; a Discovery Grant (RGPIN-2014-04465) awarded by the Natural Sciences and Engineering Research Council of Canada to CH; the Canada Research Chairs program and a Discovery Grant (RGPIN-2015-04854) awarded by the Natural Sciences and Engineering Research Council of Canada to KJ; a career development award by the Knights Templar Eye Foundation, a research grant by the Whitehall Foundation and a Sloan Research Fellowship to AM; a research grant by the Brain and Behavior Research Foundation (23017) and Whitehall Foundation to YS. The views presented in this work do not necessarily reflect those of the National Institutes of Health.

### Conflict of interest statement

The authors declare that the research was conducted in the absence of any commercial or financial relationships that could be construed as a potential conflict of interest.
